# Novel Nonsymmetrical 1,4-Dihydropyridines as Inhibitors of Nonsymmetrical MRP-Efflux Pumps for Anticancer Therapy

**DOI:** 10.3390/ph13070146

**Published:** 2020-07-09

**Authors:** David Kreutzer, Christoph A. Ritter, Andreas Hilgeroth

**Affiliations:** 1 Institute of Pharmacy, Research Group of Drug Development, Martin Luther University Halle-Wittenberg, 06108 Halle (Saale), Germany; david.kreutzer94@web.de; 2 Institute of Pharmacy, Department of Clinical Pharmacy, University of Greifswald, 17489 Greifswald, Germany; ritter@uni-greifswald.de

**Keywords:** anticancer drug resistance, structure activity, synthesis, inhibition, substituent

## Abstract

Cancer is a strong global burden with increasing numbers of diseases and ongoing anticancer drug resistance. The number of structurally novel anticancer drugs is strongly limited. They cause high costs for the social health systems. Most critical so-called multidrug resistances (MDR) are caused by transmembrane efflux pumps that transport drugs with various structures out of the cancer cells. Multidrug resistance proteins (MRPs) type 1 and 2 are found overexpressed in various kinds of cancer. There is a strong need for inhibitors of those efflux pumps. We developed novel nonsymmetrical 1,4-dihydropyridines as novel inhibitors of cancer relevant MRP types 1 and 2. The structure-dependent activities of the differently substituted derivatives were evaluated in cellular assays of respective cancer cells and are discussed. Promising candidates were identified. One candidate was demonstrated to resensitize a cisplatin resistant cancer cell line and thus to overcome the anticancer drug resistance.

## 1. Introduction

Beside cardiovascular diseases cancer is the most threatening global disease that causes millions of deaths per year [1,2,3]. Moreover, the number of diseases is expected to increase to 20 million until 2025 [3]. Early on, effective drugs were developed that were used for therapeutic treatment in cases when cure by surgery as best method failed or was not a promising option [1,3]. Those early chemotherapeutic drugs act as DNA alkylating and intercalating agents, such as topoisomerase inhibitors or antimetabolites to affect all dividing cells including healthy cells with resulting strong side effects [1]. The understanding of cancer as multifactorial disease with deregulated cellular pathways helped to identify certain potential target structures that were addressed by novel drugs in specific drug-targeted therapies [1]. Innovations in that field have been monoclonal antibodies that address cellular receptors and protein kinase inhibitors [1]. However, target structure changes by gene mutations led to resistances in many cases and to the development of multikinase inhibitors [4]. Therefore, in many cases treatment of cancer is still carried out by the use of the most effective early chemotherapeutic agents. 

Most critical resistances have been those that affect various anticancer drugs as so-called multidrug resistance (MDR) [3,5]. In case of such an MDR, only structurally novel drugs can be used. However, the number of structurally novel anticancer drugs introduced in the last decade is small as drug development mainly focused on monoclonal antibodies or structurally related protein kinase inhibitors [1]. Thus, drug development efforts need to concentrate also on causes of MDR as prospective anticancer treatment. 

The main cause of MDR is the overexpression of transmembrane efflux pumps in the course of anticancer treatment [3,6]. Mostly, the anticancer drug induces the overexpression of a respective efflux pump that transports the drug out of the cells so that the intracellular drug levels are insufficient to reach the desired anticancer effect [3,7]. Subsequently, the cancer cells become resistant towards the respective drug. Binding sites at the efflux pumps are discussed that allow different anticancer substrates to bind. Hence, the resistance mostly includes several anticancer drugs with variable structures as MDR [8]. In case of such an induced resistance the only possibility to overcome the resistance is the inhibition of the efflux pumps’ activity in order to increase the intracellular anticancer drug concentration and thus resensitize the cancer cells towards therapy [9].

We developed novel 1,4-dihydropyridines as innovative inhibitors of the multidrug resistance protein (MRP) type transporters, namely MRP1 and 2 which play the most prominent role in anticancer drug resistance within the MRP family [8]. Early 1,4-dihydropyridines of the nifedipine-type in use as antihypertensive agents have been discovered to show inhibitory effects towards the efflux pump P-glycoprotein [10]. Both early inhibitors and P-glycoprotein own a symmetrical molecular framework as will be discussed. Therefore, we developed novel non-symmetrical 1,4-dihydropyridines to investigate them as inhibitors of the non-symmetrical MRP types 1 and 2. They have been evaluated in cancer-derived cellular test systems by the use of a fluorescent substrate that was measured to prove the inhibition effect by an increase of that substrate in the cells. Structure-dependent activities of the compounds to enhance the substrate uptake by inhibiting the efflux pump are discussed. The most promising candidate was investigated in an efflux pump-overexpressing cell line. This drug candidate was able to reverse efflux pump driven anticancer drug resistance by restoring the respective drug sensitivity. 

## 2. Results and Discussion

### 2.1. Synthesis of the 1,4-Dihydropyridines

The classical 1,4-dihydropyridine formations compose the molecular scaffold which is generated by the reaction of three components, namely a mostly aromatic aldehyde, a dicarbonyl function, and an amino compound, preferably ammonia [11,12,13]. By formation of certain intermediates, the final reaction leads to symmetric compounds. Most variants have been those with different substitutions in the symmetric 2- and 6-positions and the 4-position. There have been early efforts to synthesize *N*-substituted 1,4-dihydropyridines by the use of both aliphatic or aromatic amines, but the outcome was poor so far due to the partially different activities of the amine compounds [14,15]. However, concerning the pharmacological properties of those symmetric 1,4-dihydropyridines there has been a strong focus on those without a nitrogen amine substitution [16].

We developed novel non-symmetrically substituted 1,4-dihydropyridines in a different approach than the known so-called one-pot reaction where the reaction mixture consists of the afore described three compounds to result in the molecular 1,4-dihydropyridine scaffold. 

**Figure 1:**
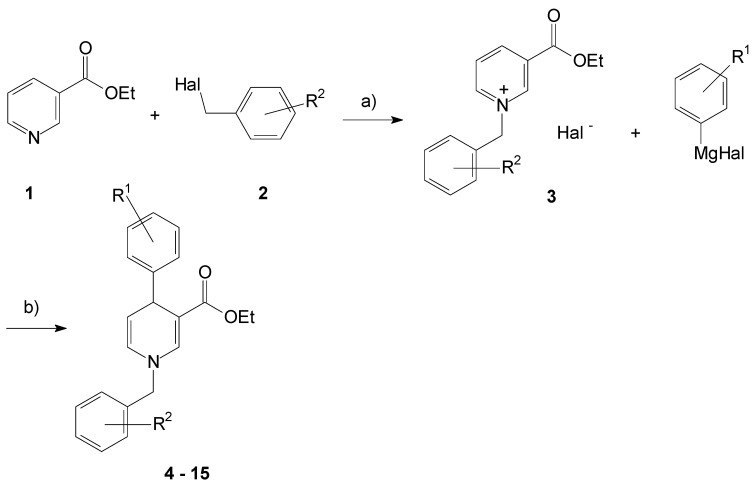
Formation of the 1,4-dihydropyridines **4**–**15**. a: 6 h, 90 °C; b: CuI, LiCl, THF, 6 h, rt.

We started with the 3-ethyl ester substituted pyridine **1** that was alkylated in the first reaction step under solvent-free conditions. The pyridine compound directly reacted with the added benzyl halogenide **2** to give the *N*-benzyl pyridinium salts **3** in good yields up to 81% after purification (Figure 1). 

Then the 4-aryl substituent was introduced by reaction of **3** with a respective Grignard reagent to be introduced as reducing reagent to generate the 1,4-dihydropyridine core. The exclusive 4-substitution of the pyridinium ring succeeded by the use of copper(I) iodide as catalyst to direct the substituent in the preferred position under mild conditions. Therefore, the non-symmetrically substituted 1,4-dihydropyridines **4**–**15** resulted in respective yields up to 61%. Spectroscopical characteristics have been the 4-protons to occur at about 4.4 ppm in the ^1^H NMR spectra, the conjugated 3-carbonyl bond at about 1680 cm^−1^, and the dihydropyridine double bond appearing at about 1580 cm^−1^ in the infrared spectra. 

### 2.2. MRP Efflux Pump Inhibition of the 1,4-Dihydropyridines

Classical 1,4-dihydropyridines with the symmetric molecular scaffold and the unsubstituted nitrogen function proved to have inhibiting properties towards P-glycoprotein as a transmembrane efflux pump that was detected in cancer cells to transport anticancer drugs out of the cells [3,17,18]. P-glycoprotein was one of the early discovered efflux pumps to be responsible for the multidrug resistance in cancer [19]. P-glycoprotein possesses a totally symmetric molecular structure of two transmembrane domains (TMDs). They each consist of six α-helical subunits that surround the *C*_2_ axis of symmetry [19,20,21]. Based on that molecular symmetry symmetric inhibitors which block the binding of an anticancer drug for the extracellular transport at the potential binding site were of favor for drug development [22].

Contrasting P-glycoprotein, the multidrug resistance associated proteins (MRPs) 1 and 2 have no symmetric structure of their TMDs and the respective α-helical subunits [8]. Moreover, they have three TMDs [8,23]. Several of those α-helical subunits have been identified in MRP1 and 2 to be involved in binding of anticancer drugs for transport, whereas the mechanism of transport is still unknown [8,23,24,25,26]. 

Inhibitors of both MRP types are rare and mostly have different pharmacological properties so that they cannot be used as inhibitors for a perspective treatment of efflux pump-derived MDR in cancer [3,8]. Moreover, they have been reported to affect various efflux pumps like P-glycoprotein, the breast-cancer resistant protein (BCRP), and MRP1 [8]. Therefore, there is also a missing selectivity for a specific efflux pump type.

We wondered whether non-symmetrical inhibitors may be of favor as MRP1 and 2 inhibitors and thus evaluated our nonsymmetrical 1,4-dihydropyridines in an in vitro assay of cells which express MRP1 and MRP2 by the use of the fluorescent carboxyfluorescein diacetate (CFDA) as MRP substrate. The respective cells were pre-incubated with the potential inhibitors at a 10 µM concentration and then the fluorescent substrate was added. The substrate uptake was measured by flow cytometry detecting the corresponding fluorescence of the respective cells. The fluorescence was related to that measured for the untreated control cells to give a fluorescence activity ratio (*FAR*) value. For determination of the MRP1 inhibiting properties of our compounds we used the ovarian carcinoma cell line A2780 which expresses MRP1 [27]. The determined *FAR* values are shown in Table 1.

In the first compound series **4**–**8** with a 4-methoxyphenyl residue in the 4-position of the 1,4-dihydropyridine core we varied the *N*-benzyl substitution with methoxy and methyl in both *meta* and *para* positions. The *meta* methoxy substitution in compound **4** resulted in a MRP1 activity that almost reached that of the MRP1 inhibitor probenecid used as standard. If moved to the *para* position in compound **5** the activity was found reduced. The *meta* methyl substituted compound **6** showed an increased activity compared to compound **4**. A movement of the methyl substituent to the *para* position reduced the activity of compound **7**. In addition, compound **7** showed a better activity than compound **5**. Thus, for that first compound series it can be concluded that a methyl substitution of the benzyl residue is more favorable than a methoxy substitution and that a *meta* substitution is more favorable than a *para* substitution.

In the second compound series **8**–**11** we used a 3-methoxyphenyl substituent and again combined that with the *N*-benzyl substitutions of methoxy and methyl residues in both *meta* and *para* positions. Compound **8** with the *meta* methoxy substitution was more active than the corresponding 4-methoxyphenyl compound **4**. A movement of the benzyl methoxy substituent to the *para* position of compound **9** led to a decrease of activity that was also observed for derivative **5**. Again, the 3-methoxyphenyl substitution was better than the 4-methoxyphenyl substitution. The *meta* methyl substitution of compound **10** resulted in an almost similar activity than that of derivative **6**. However, if moved to the *para* position in compound **11** we found an increased activity if compared to compound **7**. This suggests that the 3-methoxyphenyl substitution is more favorable than the 4-methoxyphenyl substitution.

In a third compound series **12**–**15** we combined the 4- and the 3-methoxy function at the 4-phenyl residue with those methoxy and methyl substitutions of the *N*-benzyl residue to check the observed structure–activity relationships of which substitutions are of favor at the given position. The *meta* methoxy benzyl substitution of compound **12** showed a similar activity compared to compounds **4** and **8**. The *para* methoxy substituted derivative **13** showed a reduced activity compared to **12** and an increased activity compared to derivatives **5** and **7**. The *meta* methyl substituted derivative **14** showed similar activity compared to compounds **6** and **10**. The *para* methyl substituted derivative **15** showed a reduced activity compared to the *meta* substituted derivative and a similar activity to derivative **7**. Interestingly in the series of dimethoxyphenyl substituted derivatives we found confirmation of the observation that the *para* benzyl substituted compounds are less active than the *meta* substituted derivatives.

Next we evaluated our three compound series against MRP2. For that purpose, the ovarian cancer cell line A2780cis was used. That cell line is characterized by the overexpression of MRP2 as a consequence of the treatment of A2780 with *cis*-platin that induced the expression of MRP2 [27]. Accumulation of the fluorescent MRP substrate CFDA in the MRP2 overexpressing cells under the used compounds treatment compared to the untreated control cells was expressed as *FAR* value. In order to differentiate the effect of the MRP2 from MRP1 inhibition a ratio was calculated of this *FAR* value and that given for the treated MRP1-expressing A2780 cells. The *FAR* values of the MRP2 overexpressing cells and the calculated MRP2/MRP1 ratios are shown in Table 1.

The MRP2 inhibition properties of our first compound series **4**–**8** with the 4-methoxyphenyl substitution at the molecular scaffold were all better than respective MRP1 inhibiting activities with best results for the methoxy benzyl substituted compounds **4** and **5**. Comparing the methoxy and the methyl substitution effects of the *N*-benzyl residue on MRP2 activity the methoxy substitutions were better than the methyl substitutions both in the *para* and the *meta* position.

The compounds of our second series **8**–**11** with the 3-methoxyphenyl substitution at the scaffold showed mostly reduced MRP2 inhibition activities compared to those with the 4-methoxyphenyl substitution. Thus, the 4-methoxyphenyl substitution was more favorable for MRP2 inhibition. Concerning the positioning of the substituent within the *N*-benzyl residue, the *para* substitution tends to be more favorable than the *meta* position.

In our third compound series **12**–**15** with the dimethoxy substitution of the 4-phenyl residue we found mostly better MRP2 than MRP1 inhibition activities. Concerning the kind of substituent at the *N*-benzyl residue the methyl substitution was better than the methoxy substitution at both the *meta* and *para* positions of the *N*-benzyl residue. Best results of MRP2 inhibition were found for the *meta* methyl substituted derivative **14** within this compound series.

### 2.3. In Vitro MRP Resistance Studies of Drug Reversal

The overexpression of efflux pumps in cancer as main cause of anticancer drug resistance correlates with increased gene levels of the respective efflux pump [8]. Therefore, various kinds of cancer have been associated with increased expression rates of MRP1 and MRP2. Those have been described for aggressive breast carcinoma and lung cancer subtypes such as non-small cell lung cancer. Increased levels of MRP1 and 2 have also been reported for colorectal and renal carcinoma patients [8]. Therefore, there is a certain influence of MRPs on the outcome of clinical cancer treatment.

Novel efflux pump inhibitors should be profiled in an MRP-overexpressing cell line to prove an effect of the efflux pump inhibition on the reversal of the efflux pump mediated anticancer drug resistance. Recently one inhibitor has been reported to show such in vitro effects [3,28]. However, first conducted in vivo studies disappointed [3]. Moreover, that inhibitor was a protein kinase inhibitor. Therefore, an exclusive role in the MRP-mediated in vitro effect was difficult to conclude due to the protein kinase inhibitory properties of the compound.

We decided to profile one of our best MRP1- and MRP2-inhibiting compounds, compound **14**, in the MRP-overexpressing ovarian carcinoma cell line A2780cis. We used the MRP substrate *cis*-platin as anticancer drug and determined the cellular toxicity of that drug in both the MRP2-overexpressing cell line A2780cis and the non-overexpressing cell line A2780. The toxicity was measured in an MTT (3-(4,5-dimethylthiazol-2-yl)-2,5-diphenyltetrazolium bromide) assay by UV absorption. This assay measures formazan production as a result of the reducing activity of the mitochondrial dehydrogenases. The determined IC_50_ values of *cis*-platin were 0.41 nM in the non-expressing cell line and 19.60 µM in the MRP2-overexpressing cell line. That means a given sensitivity of *cis*-platin in the non-expressing cell line and a loss of sensitivity in the MRP2-overexpressing cell line.

First, we used the MRP inhibitor probenecid to investigate a potential influence on the reversal of the MRP2-mediated resistance towards the used drug *cis*-platin. A used concentration of 10 µM of probenecid led to an IC_50_ value of 12.65 µM for *cis*-platin in the MRP2-overexpressing cell line. Therefore, there was only a small effect in the increase of the anticancer drug toxicity. Next we used one of our best MRP inhibiting agents, compound **14**, and determined the effect of 10 µM of that inhibitor on the *cis*-platin drug toxicity. We determined an IC_50_ value of 8.15 nM for *cis*-platin in the MRP2-overexpressing cell line. That means a strong effect on the toxicity that almost reached that of *cis*-platin in the non-expressing cell line and a reversal of the MRP-mediated anticancer drug resistance.

## 3. Materials and Methods

### 3.1. Chemical Reagents and Instruments

Commercial reagents were used without further purification. The ^1^H-NMR spectra (500 MHz) were measured using tetramethylsilane as internal standard. Thin layer chromatography (TLC) was performed on E. Merck 5554 silica gel plates. The high-resolution mass spectra were recorded on a Finnigan LCQ Classic mass spectrometer. IR spectra were recorded on a FT-IR spectrometer.

### 3.2. General Procedure for the Synthesis of Compound ***3***

One equivalent of nicotinic acid ethyl ester **1** was heated in a round flask at 90 °C. At that temperature one and a half equivalent of the respective benzyl halogenide **2** was added dropwise under stirring. The reaction proceeding was followed by TLC. After finishing of the reaction, the mixture was left cooling to room temperature. Then it was dissolved in a water/methanol mixture (1/1) and extracted with chloroform. Then the methanol/water phase was treated with toluene for several times and each volume was reduced in vacuum. Then under addition of acetone and cooling the benzylated nicotinic acid ester compounds **3** crystallized as pale yellow powders and were finally stored over phosphorus pentoxide.

### 3.3. General Procedure for the Synthesis of Compounds ***4***–***15***

One equivalent of the respective compound **3** was suspended in dried THF (5 mL) at room temperature under argon atmosphere. After addition of 0.1 equivalent of copper (I) iodide and 0.2 equivalent of lithium chloride eight equivalents of the respective Grignard reagent used as 1.0 M solution in THF were added dropwise under stirring. The reaction proceeding was followed by TLC. After finishing of the reaction, a solution of ammonium chloride (20%) was added and the mixture was extracted with chloroform for three times. The organic layer was then extracted with a mixture of ammonium chloride (20%) and concentrated ammonia (1/1), a hydrochloric acid solution (10%), and a saturated sodium chloride solution. Finally, the organic layer was dried over sodium sulfate and filtered. Then the organic layer was removed in vacuum and the remaining oily product was purified by column chromatography using silica gel and a mixture of cyclohexane and acetic acid ethyl ester (4/1) as eluent to result in compounds **4**–**15** that were stored at low temperature. The purity of the compounds was >95% by NMR and was further evaluated by elemental analysis for the most active derivatives **4**, **5***,* **8**, **11**, and **14**.

*Ethyl N-(3-methoxybenzyl)-4-(4-methoxyphenyl)-1,4-dihydropyridine-3-carboxylate (4)*. Yield 45%, yellow liquid; ^1^H NMR (methanol-*d*_4_) *δ* = 7.28–7.23 (m, 1H, 5′-H), 7.15–7.09 (m, 2H, 2″-, 5″-H), 6.86 (s, 1H, 2–H), 6.84–6.66 (m, 5H, 2″-, 4′-, 6′-, 3″-, 5″-H), 6.01 (d, *J* = 7.8 Hz, 1H, 6-H), 4.89 (dd, *J* = 7.8, 4.9 Hz, 1H, 5-H), 4.47 (s, 2H, CH_2_), 4.38 (d, *J* = 4.9 Hz, 1H, 4-H), 4.00 (qd, *J* = 7.1, 5.2 Hz, 2H, CH_2_CH_3_), 3.80 (s, 3H, OCH_3_), 3.74 (s, 3H, OCH_3_), 1.13 (t, *J* = 7.1 Hz, 3H, CH_2_CH_3_); *m/z* (ESI) 402.47 (M+Na^+^); IR (cm^−1^) = 2926 (m, C–H aliph.), 2833 (m, C-H aliph), 1680 (m, C=O), 1584 (s, C=C). Calcd. for C_23_H_25_NO_4_ (%): C 72.80, H 6.64, N 3.69; Found: C 72.40, H 6.45, N 3.35.

*Ethyl N-(4-methoxybenzyl)-4-(4-methoxyphenyl)-1,4-dihydropyridine-3-carboxylate (5)*. Yield 29%, yellow liquid; ^1^H NMR (methanol-*d*_4_) *δ* = 7.24 (dd, *J* = 8.8, 2.9 Hz, 2H, 2″-, 6″-H), 7.08 (d, *J* = 8.8 Hz, 2H, 2′-, 6′-H), 6.93 (s, 1H, 2–H), 6.73 (dd, *J* = 8.8, 29 Hz, 4H, 3′-, 5′-, 3″-, 5″-H), 6.00 (d, *J* = 7.8 Hz, 1H, 6-H), 4.87 (dd, *J* = 7.8, 4.9 Hz, 1H, 5-H), 4.42 (s, 2H, CH_2_), 4.36 (d, *J* = 4.9 Hz, 1H, 4-H), 3.99 (qd, *J* = 7.1, 4.7 Hz, 2H, CH_2_CH_3_), 3.80 (s, 3H, OCH_3_), 3.74 (s, 3H, OCH_3_), 1.13 (t, *J* = 7.1 Hz, 3H, CH_2_CH_3_); *m/z* (ESI) 402.58 (M+Na^+^); IR (cm^−1^) = 2929 (w, C–H aliph.), 2833 (w, C–H aliph), 1686 (w, C=O), 1584 (s, C=C). Calcd. for C_23_H_25_NO_4_ (%): C 72.80, H 6.64, N 3.69; Found: C 72.55, H 6.56, N 3.45.

*Ethyl 4-(4-methoxyphenyl)-N-(3-methylbenzyl)-1,4-dihydropyridine-3-carboxylate (6)*. Yield 12%, yellow liquid; ^1^H NMR (methanol-*d*_4_) *δ* = 7.29–7.27 (m, 1H, 5′-H), 7.17 (m, 5H, 2′-, 4′-, 6′-, 2″-, 6″-H), 7.08–7.04 (m, 1H, 2–H), 6.99–6.90 (m, 2H, 3″-, 5″-H), 6.07–6.03 (m, 1H, 6-H), 4.93 (dd, *J* = 7.8, 4.9 Hz, 1H, 5-H), 4.51 (s, 2H, CH_2_), 4.42 (d, *J* = 4.9 Hz, 1H, 4-H), 4.04 (qd, *J* = 7.1, 5.0 Hz, 2H, CH_2_CH_3_), 3.79 (s, 3H, OCH_3_), 2.40 (s, 3H, CH_3_), 1.18 (t, *J* = 7.1 Hz, 3H, CH_2_CH_3_); *m/z* (ESI) 386.81 (M+Na^+^); IR (cm^−1^) = 2926 (w, C-H aliph.), 1680 (m, C=O), 1589 (s, C=C).

*Ethyl 4-(4-methoxyphenyl)-N-(4-methylbenzyl)-1,4-dihydropyridine-3-carboxylate (7)*. Yield 11%, yellow liquid; ^1^H NMR (methanol-*d*_4_) *δ* = 7.24 (m, 4H, 2′-, 3′-, 5′, 6′-H), 7.16–7.13 (m, 2H, 2″-, 6″-H), 6.95–6.89 (m, 1H, 2–H), 6.84–6.79 (m, 2H, 3″-, 5″-H), 6.04 (dd, *J* = 7.8, 1.6 Hz, 1H, 6-H), 4.92 (dd, *J* = 7.8, 4.8 Hz, 1H, 5-H), 4.49 (s, 2H, CH_2_), 4.41 (d, *J* = 4.8 Hz, 1H, 4-H), 4.04 (qd, *J* = 7.1, 4.7 Hz, 2H, CH_2_CH_3_), 3.79 (s, 3H, OCH_3_), 2.39 (s, 3H, CH_3_), 1.17 (t, *J* = 7.1 Hz, 3H, CH_2_CH_3_); *m/z* (ESI) 386.85 (M+Na^+^); IR (cm^−1^) = 2929 (w, C–H aliph.), 1680 (m, C=O), 1585 (s, C=C).

*Ethyl N-(3-methoxybenzyl)-4-(3-methoxyphenyl)-1,4-dihydropyridine-3-carboxylate (8)*. Yield 61%, yellow liquid; ^1^H NMR (methanol-*d*_4_) *δ* = 7.29–7.22 (m, 1H, 5′-H), 7.12 (t, *J* = 7.8 Hz, 1H, 5″-H), 6.91–6.74 (m, 7H,2-, 2′-, 4′-, 6′-, 2″-, 4″-, 6″-H), 6.03 (dd, *J* = 7.9, 1.6 Hz, 1H, 6-H), 4.91 (dd, *J* = 7.9, 4.9 Hz, 1H, 5-H), 4.48 (s, 2H, CH_2_), 4.42 (d, *J* = 4.9 Hz, 1H, 4-H), 4.00 (qd, *J* = 7.1, 5.6 Hz, 2H, CH_2_CH_3_), 3.79 (s, 3H, OCH_3_), 3.70 (s, 3H, OCH_3_), 1.13 (t, *J* = 7.1 Hz, 3H, CH_2_CH_3_); *m/z* (ESI) 402.58 (M+Na^+^); IR (cm^−1^) = 2914 (w, C-H aliph.), 2848 (w, C-H aliph), 1686 (s, C=O), 1586 (s, C=C). Calcd. for C_23_H_25_NO_4_ (%): C 72.80, H 6.64, N 3.69; Found: C 72.56, H 6.57, N 3.48.

*Ethyl N-(4-methoxybenzyl)-4-(3-methoxyphenyl)-1,4-dihydropyridine-3-carboxylate (9)*. Yield 29%, yellow liquid; ^1^H NMR (methanol-*d*_4_) *δ* = 7.29–7.20 (m, 2H, 2′-, 6′-H), 7.14–7.10 (m, 1H, 5″-H), 6.94 (s, 1H, 2H), 6.92–6.90 (m, 2H, 3′-, 5′-H), 6.80–6.65 (m, 3H, 2″-, 4″-, 6″-H), 6.02 (dd, *J* = 7.8, 1.6 Hz, 1H, 6-H), 4.89 (dd, *J* = 7.8, 4.9 Hz, 1H, 5-H), 4.43 (s, 2H, CH_2_), 4.40 (d, *J* = 4.9 Hz, 1H, 4-H), 4.00 (qd, *J* = 7.1, 5.2 Hz, 2H, CH_2_CH_3_), 3.79 (s, 3H, OCH_3_), 3.69 (s, 3H, OCH_3_), 1.12 (t, *J* = 7.1 Hz, 3H, CH_2_CH_3_); *m/z* (ESI) 402.62 (M+Na^+^); IR (cm^−1^) = 2915 (w, C-H aliph.), 2849 (w, C-H aliph), 1683 (s, C=O), 1584 (s, C=C).

*Ethyl N-(3-methylbenzyl)-4-(3-methoxyphenyl)-1,4-dihydropyridine-3-carboxylate (10)*. Yield 17%, yellow liquid; ^1^H NMR (methanol-*d*_4_) *δ* = 7.25 (t, *J* = 7.4 Hz, 1H, 5′-H), 7.13–7.11 (m, 4H, 2′-, 4′-, 6′-, 5″-H), 6.93–6.86 (m, 1H, 2–H), 6.83–6.66 (m, 3H, 2″-, 4″-, 6″-H), 6.04–6.00 (m, 1H, 6-H), 4.90 (dd, *J* = 7.8, 4.9 Hz, 1H, 5-H), 4.47 (s, 2H, CH_2_), 4.42 (d, *J* = 4.9 Hz, 1H, 4-H), 4.00 (qd, *J* = 7.1, 5.3 Hz, 2H, C**H**_2_CH_3_), 3.70 (s, 3H, OCH_3_), 2.35 (s, 3H, CH_3_), 1.13 (t, *J* = 7.1 Hz, 3H, CH_2_C**H**_3_); *m/z* (ESI) 386.26 (M+Na^+^); IR (cm^−1^) = 2926 (w, C-H aliph.), 1682 (m, C=O), 1584 (s, C=C).

*Ethyl N-(4-methylbenzyl)-4-(3-methoxyphenyl)-1,4-dihydropyridine-3-carboxylate (11)*. Yield 40%, yellow liquid; ^1^H NMR (methanol-*d*_4_) *δ* = 7.24 (m, 3H, 2′-, 6′-, 5″-H), 7.19–7.13 (m, 2H, 3′-, 5′-H), 6.93–6.89 (m, 1H, 2–H), 6.85-6.70 (m, 3H, 2″-, 3″-, 6″-H), 6.06 (dd, *J* = 7.8, 1.6 Hz, 1H, 6-H), 4.94 (dd, *J* = 7.8, 4.9 Hz, 1H, 5-H), 4.50 (s, 2H, CH_2_), 4.46 (d, *J* = 4.8 Hz, 1H, 4-H), 4.05 (qd, *J* = 7.1, 5.1 Hz, 2H, C**H**_2_CH_3_), 3.74 (s, 3H, OCH_3_), 2.38 (s, 3H, CH_3_), 1.17 (t, *J* = 7.1 Hz, 3H, CH_2_C**H**_3_); *m/z* (ESI) 386.74 (M+Na^+^); IR (cm^−1^) = 2926 (w, C-H aliph.), 1683 (m, C=O), 1585 (m, C=C). Calcd. for C_23_H_25_NO_3_ (%): C 76.01, H 6.93, N 3.85; Found: C 75.75, H 6.65, N 3.54.

*Ethyl 4-(3,4-dimethoxyphenyl)-N-(3-methoxybenzyl)-1,4-dihydropyridine-3-carboxylate (12)*. Yield 19%, yellow liquid; ^1^H NMR (methanol-*d*_4_) *δ* = 7.34 (t, *J* = 3.7 Hz, 1H, 5′-H), 7.01-6.94 (m, 4H, 2-, 2′-, 4′-, 6′-H), 6.82–6.77 (m, 3H, 2″-, 5″-, 6″-H), 6.13–6.07 (m, 1H, 6-H), 4.95 (dd, *J* = 7.8, 4.9 Hz, 1H, 5-H), 4.52 (s, 2H, CH_2_), 4.43 (d, *J* = 4.9 Hz, 1H, 4-H), 4.06 (qd, *J* = 7.1, 5.2 Hz, 2H, C**H**_2_CH_3_), 3.81 (s, 6H, 2 x OCH_3_), 3.74 (s, 3H, OCH_3_), 1.19 (t, *J* = 7.1 Hz, 3H, CH_2_C**H**_3_); *m/z* (ESI) 432.45 (M+Na^+^); IR (cm^−1^) = 2930 (m, C-H aliph.), 2835 (w, OCH_3_), 1677 (m, C=O), 1586 (m, C=C).

*Ethyl 4-(3,4-dimethoxyphenyl)-N-(4-methoxybenzyl)-1,4-dihydropyridine-3-carboxylate (13)*. Yield 3%, yellow liquid; ^1^H NMR (methanol-*d*_4_) *δ* = 7.30 (d, *J* = 8.5 Hz, 2H, 2′-, 6′-H), 7.05 (s, 1H, 2–H), 6.95–6.72 (m, 5H, 3′-, 5′-, 2″-, 5″-, 6″-H), 6.12-6.06 (m, 1H, 6-H), 4.93 (dd, *J* = 7.8, 4.9 Hz, 1H, 5-H), 4.45 (s, 2H, CH_2_), 4.41 (d, *J* = 4.9 Hz, 1H, 4-H), 4.05 (qd, *J* = 7.1, 4.1 Hz, 2H, C**H**_2_CH_3_), 3.88 (s, 6H, 2 x OCH_3_), 3.71 (s, 3H, OCH_3_), 1.19 (t, *J* = 7.1 Hz, 3H, CH_2_C**H**_3_); *m/z* (ESI) 432.88 (M+Na^+^); IR (cm^-1^) = 2936 (w, C-H aliph.), 2837 (w, OCH_3_), 1668 (m, C=O), 1592 (w, C=C).

*Ethyl 4-(3,4-dimethoxyphenyl)-N-(3-methylbenzyl)-1,4-dihydropyridine-3-carboxylate (14)*. Yield 32%, yellow liquid; ^1^H NMR (methanol-*d*_4_) *δ* = 7.32–7.29 (m, 1H, 5′-H), 7.06 (m, 3H, 2H-, 4′-, 6′-H), 6.97 (s, 1H, 2–H), 6.87–6.78 (m, 3H, 2″-, 5″-, 6″-H), 6.09 (d, *J* = 7.9 Hz, 1H, 6-H), 4.95 (dd, *J* = 7.9, 4.9 Hz, 1H, 5-H), 4.52 (s, 2H, CH_2_), 4.42 (d, *J* = 4.9 Hz, 1H, 4-H), 4.06 (qd, *J* = 7.1, 5.2 Hz, 2H, C**H**_2_CH_3_), 3.82 (s, 3H, OCH_3_), 3.72 (s, 3H, OCH_3_), 2.39 (s, 3H, CH_3_), 1.19 (t, *J* = 7.1 Hz, 3H, CH_2_C**H**_3_); *m/z* (ESI) 393.64 (M^+^); IR (cm^–1^) = 2925 (m, C-H aliph.), 2850 (w, OCH_3_), 1680 (m, C=O), 1571 (m, C=C). Calcd. for C_24_H_27_NO_4_ (%): C 73.26, H 6.92, N 3.56; Found: C 72.85, H 6.68, N 3.33.

*Ethyl 4-(3,4-dimethoxyphenyl)-N-(4-methylbenzyl)-1,4-dihydropyridine-3-carboxylate (**15**)*. Yield 14%, yellow liquid; ^1^H NMR (methanol-*d*_4_) *δ* = 7.17 (dd, *J* = 8.0, 1.0 Hz, 4H, 2′-, 3′-, 5′-, 6′-H), 6.91 (s, 1H, 2–H), 6.87–6.75 (m, 3H, 2″-, 5″-, 6″-H), 6.09 (d, *J* = 7.8 Hz, 1H, 6-H), 4.93 (dd, *J* = 7.9, 4.9 Hz, 1H, 5-H), 4.50 (s, 2H, CH_2_), 4.41 (d, *J* = 4.9 Hz, 1H, 4-H), 4.05 (qd, *J* = 7.1, 5.0 Hz, 2H, C**H**_2_CH_3_), 3.82 (s, 3H, OCH_3_), 3.72 (s, 3H, OCH_3_), 2.38 (s, 3H, CH_3_), 1.19 (t, *J* = 7.1 Hz, 3H, CH_2_C**H**_3_); *m/z* (ESI) 416.94 (M^+^); IR (cm^−1^) = 2926 (m, C-H aliph.), 2850 (w, OCH_3_), 1680 (m, C=O), 1570 (m, C=C).

### 3.4. MRP Inhibition Assay

The ovarian carcinoma cell lines A2780 and A2780cis were obtained from PD Dr. Dr. Hermann Lage from the Virchow hospital, Berlin, Germany. Cells were cultured in RPMI-1640 medium that was supplemented with fetal bovine serum (10%) and penicillin/streptomycin (1%) at 37 °C and under carbon dioxide atmosphere (5%). To ensure continuous MRP2 expression, the cell line A2780cis was additionally cultured with *cis*-platin (1 µM).

In the assay each 500,000 cells were centrifuged with 2000 UpM at 4 °C in an Eppendorf tube. The supernatant was removed, and the samples were stored on ice. Then they were resuspended in RPMI-1640 medium and test compounds and the probenecid control were added from stock solutions in DMSO (dimethyl sulfoxide) to give a final concentration of 10 µM. The samples were cultured at 37 °C and 1200 UpM in a thermomixer. Then the fluorescent CFDA was added from a PBS solution to give a final concentration of 1 µM. The samples were centrifuged again, and the supernatant was removed. Then, PBS was added, and the samples were centrifuged again. That washing procedure was repeated. Finally, the fluorescence of the resuspended cells was measured by flow cytometry using 10,000 cells and a MACSQuant Analyzer. The measurement was conducted each for three times in inhibitor treated and untreated control cells. The *FAR* values were calculated by division of the fluorescence of the treated to the untreated control cells.

### 3.5. MRP Reversal Assay

A total of 10,000 cells of each cell line were cultured in wells of a 96-well plate at 37 °C under a carbon dioxide atmosphere (5%). Then increasing concentrations of *cis*-platin from 0.01 µM to finally 20 µM, the test compound and probenecid were added, respectively. The plate was incubated for 48 h under the origin culture conditions. Then the MTT reagent was added to each well (10 µL of a stock solution of 5 mg/mL in PBS) and incubation continued for 4 h. Then 100 µL DMSO was added to each well to solve the formazan reduction product. The plate was shaken for 30 min on a plate shaker and finally the formazan absorption was measured. The described method was repeated for three times. The IC_50_ values were determined from the resulting sigmoid curves.

## 4. Conclusions

Anticancer therapies suffer from the ongoing multidrug resistance (MDR) against various drugs. In case of such an MDR the number of alternatives is low, because only structurally different anticancer drugs may not be affected by the MDR phenomenon. Structurally novel drugs have been rare in the last decade and are mostly used only in case of a special kind of cancer in the so-called targeted therapies. An alternative would be a more general approach to reverse MDR. Mostly, MDR is mediated by transmembrane efflux pumps. An inhibition of their activity is a strategy to combat MDR. Inhibitors of such efflux pumps have been searched for, but investigated compounds have other pharmacological activities so that their use is not possible in anticancer drug therapies. We developed novel 1,4-dihydropyridines as non-symmetrical compounds that have been investigated to inhibit MRP1 and MRP2, both with a non-symmetric framework. Within our compound series we identified favorable substitution patterns such as 3-methoxyphenyl substituents and *meta N*-benzyl substituents for the inhibition of MRP1 and 4-methoxyphenyl substituents and *para N*-benzyl substituents for the inhibition of MRP2. One compound with both favorable MRP1 and MRP2 inhibiting properties was evaluated to reverse the MDR in an overexpressing cell model. The compound was effective to resensitize the cancer cell towards *cis*-platin as MRP substrate with almost the same toxicity of *cis*-platin compared to the non-MRP-expressing cell line. Thus, we demonstrated the proof-of-principle for an inhibition of the efflux pumps to reverse MDR caused by efflux pump activity. The results encourage to follow the strategy to combat MDR-mediated cancer by such novel inhibitors.

## Figures and Tables

**Table 1:** Multidrug resistance-associated protein (MRP)1 and MRP2 inhibition data of target compounds **4**–**15** expressed as *FAR* values and calculated ratios.

*FAR* Value ^a^					
Cpd.	R^1^	R^2^	MRP1	MRP2	Ratio MRP2/MRP1
**4**	4-OMe	3-OMe	1.07 ± 0.17	2.01 ± 0.23	1.88
**5**	4-OMe	4-OMe	0.81 ± 0.15	1.40 ± 0.26	1.75
**6**	4-OMe	3-Me	1.16 ± 0.18	1.32 ± 0.32	1.13
**7**	4-OMe	4-Me	1.04 ± 0.11	1.55 ± 0.34	1.49
**8**	3-OMe	3-OMe	1.35 ± 0.04	1.33 ± 0.32	0.99
**9**	3-OMe	4-OMe	1.08 ± 0.13	1.59 ± 0.31	1.47
**10**	3-OMe	3-Me	1.12 ± 0.12	1.46 ± 0.34	1.30
**11**	3-OMe	4-Me	1.50 ± 0.19	1.57 ± 0.31	1.05
**12**	3,4-OMe	3-OMe	1.32 ± 0.13	1.48 ± 0.37	1.17
**13**	3,4-OMe	4-OMe	1.31 ± 0.17	1.13 ± 0.21	0.86
**14**	3,4-OMe	3-Me	1.17 ± 0.19	1.78 ± 0.43	1.52
**15**	3,4-OMe	4-Me	1.00 ± 0.09	1.39 ± 0.32	1.39
Probenecid			1.23 ± 0.11	1.00 ± 0.21	0.86

^a^ Mean of three determinations.
